# The Outcome of Ipsilateral Hemihepatectomy in Mucin-Producing Bile Duct Tumors

**DOI:** 10.1371/journal.pone.0092010

**Published:** 2014-04-11

**Authors:** Xin-wei Yang, Jue Yang, Liang Li, Xing-zhou Yan, Bao-hua Zhang, Feng Shen, Meng-chao Wu

**Affiliations:** Eastern Hepatobiliary Surgery Hospital, Second Military Medical University, Shanghai, China; University of Modena & Reggio Emilia, Italy

## Abstract

**Background:**

Mucin-producing bile duct tumors (MPBTs) are unusual, and we present our experience with nine surgically proven cases.

**Methods:**

Between November 2002 and November 2012, 9 patients with surgically proven MPBTs (including history of relevant hepatobiliary surgery in 6 patients) were encountered. Their clinical, imaging, and surgical findings were reviewed.

**Results:**

The most common symptom is intermittent jaundice, which occurs in seven patients. The diagnostic specificity was 77.8% by preoperative Magnetic Resonance Cholangiopancreatography (MRCP). All the patients underwent ipsilateral hemihepatectomy or remnant hemihepatectomy, accompanied caudate lobectomy in one case and concomitant Roux-en-Y choledochojejunostomy in four cases. Postoperative course was uneventful. One patient, who had intra-abdominal recurrence 59 months after surgery, was received reoperation without recurrence at the last follow-up. The remaining eight patients were alive without disease recurrence.

**Conclusion:**

Based on our follow up of 9 cases that were surgically treated for MPBTs, we conclude that ipsilateral hemihepatectomy is a safe surgical procedure with an observed recurrence risk of 11.1% and all long-term survival.

## Introduction

Most tumors arising from the intra- or extrahepatic bile ducts have the capacity to produce mucin, which is retained in the tumor cells in most cases. However, some tumors produce copious mucin within the intra- or extrahepatic bile ducts, resulting in obstructive jaundice and cholangitis, as well as marked dilatation of bile ducts. These tumors have been reported as mucin-producing bile duct tumors [1].

MPBTs have been the subject of recent attention duing to its peculiar histopathology, biological and clinical behavior, varied radiological manifestations, good prognosis [2]–[4], and histopathological similarity to intraductal papillary mucinous neoplasm of pancreas (IPMN) [5], [6]. In clinical work, MPBTs are often mistaken for biliary lithiasis because mucous is highly viscous [7]. Because of unaccurately preoperative diagnosis, surgeron usually failed to determine the initiation site of tumor, as well as the tumor invasion range in superficial mucosal layer. Such delay of definitive diagnosis and receiving biliary surgery without curative resection of primary lesion would make patients' prognosis poor and add patients' financial burden [1].

In this study, preoperative imaging characteristics (including MRCP performance), exact range of liver resection, incidence of postoperative complications and long-term survival were retrospectively analyzed. The purpose of this study is to assess the safety and outcome of ipsilateral hemihepatectomy in MPBTs. To our knowledge, this is the largest case series of this unusual biliary tumor in literature.

### Ethics

All studies were approved by the Committee on Ethics of Second Military Medical University. Every written consent was given by the patients for their information to be stored in hospital database and used for research on admission.

### Diagnostic criteria

**Figure 1:**
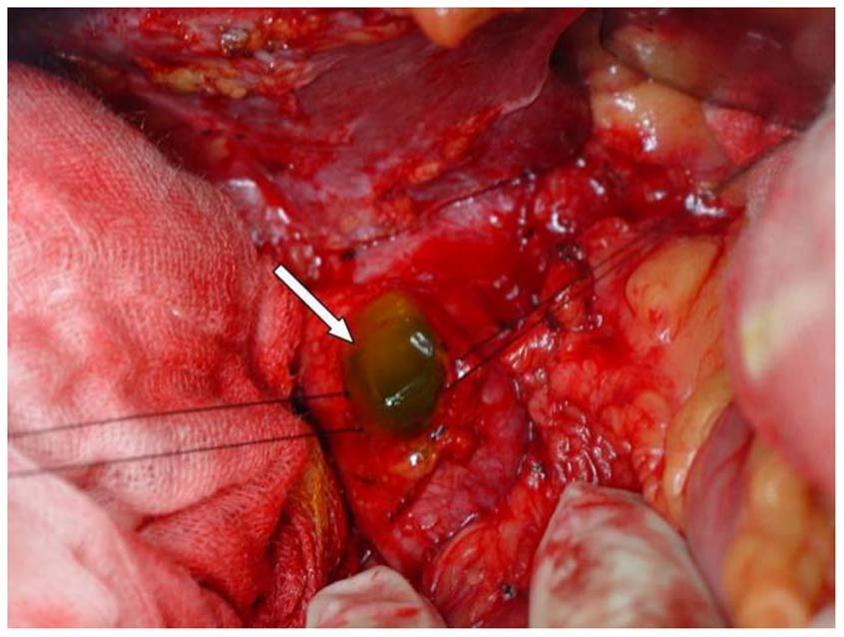
Intra-operative situs. Shown: Intraductal mucin was effused after incision of common bile duct.

The preoperative diagnostic criteria for MPBTs is descriped as follows [8]: (1) radiographic fluid retention in cystic lesion and/or dilated bile ducts; (2) confirmation of the fluid as mucin by percutaneous transhepatic biliary drainage and/or endoscopic retrograde cholangiography and/or surgical specimens (Fig. 1); (3) development from intrahepatic or extrahepatic bile ducts; and (4) cystic formation and/or dilatation of bile ducts. The final diagnoses are identified by pathological investigation.

## Patients and Methods

Between November 2002 and November 2012 , nine surgically proven MPBTs were given resection with curative intent in our hospital. The patients' medical records were reviewed for clinical manifestations, past known diseases, and available laboratory data pertaining to their condition. All patients had undergone preoperative chest radiographs and abdominal ultrasonography. There were six CT and nine MRCP imaging studies (including 6 patients underwent both CT and MR studies), with the results being available for analysis. The imaging findings were reviewed and special attention was given to the following findings: tumor size, margin, location, dilatation of the intra- and extrahepatic bile ducts, presence of effusion, CT density and enhancement value, inherent MR signal intensities, and morphologic characteristics of the tumors.

The surgical findings, including an investigation of tumor origin and cancer extension, surgical methods adopted, blood loss during surgery, and the presence of any adhesion or invasion of adjacent structures, were recorded. The pathologic examinations were reviewed by experienced pathologists. All the 9 patients were available for long-term assessment. For patients, postoperative survival time and status of recurrence were recorded. The median follow-up of all patients was 92 months (range from 6 months to 125 months). Patients were followed up regularly in outpatient clinics at 3-month intervals by undergoing blood test, ultrasonography, and computed tomography for up to 5 years after surgery. Information after 5 years was collected by telephone or personal interview.

## Results

The clinical and imaging findings of the patients were summarized in Table 1. The operative findings and follow-up were summarized in Table 2.

**Table 1:** Summary of Clinical and Imaging Features of Nine Cases of MPBTs.

Patient No.	Age(years)/gender	Presentations	History of relevant hepatobiliary surgery	MRCP Findings
1	60/M	IJ, AC	Yes(CE+BDE)	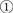 + 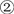 + 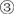 + 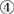
2	62/F	IJ, Fever	Yes(CE+LLH+BDE)	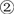 + 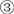 + 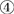 + 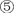
3	45/M	IJ, Fever	No	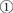 + 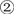 + 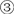 + 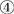 + 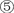
4	66/M	IJ, Fever	No	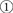 + 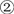 + 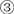
5	62/M	AC, Debilitation	No	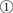 + 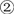 + 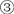 + 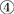 + 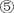
6	48/F	IJ	Yes(CE+BDE)	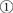 + 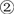 + 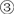 + 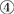
7	57/F	IJ, AC	Yes(CE+BDE)	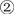 + 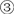 + 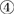 + 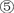
8	56/M	AC	Yes(CE+LLH+BDE)	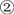 + 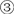 + 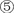
9	65/M	IJ	Yes(CE+BDE)	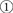 + 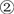 + 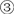 + 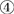

IJ: Intermittent jaundice; AC: Abdomal discomfort; CE: cholecystectomy; BDE: bile duct exploration; LLH: left lateral hepatectomy; MRCP Findings: 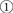 On T2 phase, showing signs of stratification between mucus and bilirubin; 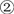 Without significantly increased gallbladder; 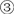
No suddenly cut-off performance of bile duct; 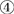 Asymmetry of bile duct dilatation; 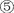 Mass performance within bile duct contrasted to bilirubin.

**Table 2:** Summary of Operative Findings and Follow-Up of Nine Cases of MPBTs.

Patient No.	Tumor Location	Operative procedure	Follow-up(no. of months after radical operation)
1	Left lateral anterior	LH+BDR+CJ	Alive, NR(6)
2	Left intrahepatic bile duct	RLH	Alive(84), Recurrence(59)
3	Hilum, right anterior superior segment	CE+LH+AH	Alive, NR (96)
4	Left intrahepatic bile duct	LH+CE	Alive, NR (108)
5	Hilum, left hepatic duct	LH+CE+AH	Alive , NR (88)
6	Left intrahepatic bile duct	LH	Alive , NR (104)
7	Left intrahepatic bile duct	LH+CE+BDR+AH+CJ	Alive, NR (104)
8	Hilum, left hepatic duct	RLH+AH+CJ	Alive , NR (78)
9	Left hepatic duct	LH+CL+BDR+CJ	Alive , NR (21)

LH: left hemihepatectomy; RLH: remnant left hemihepatectomy; BDR: bile duct resection; AH: anaplasty of hilar bile duct; CJ: choledochojejunostomy; CE: cholecystectomy; CL: caudate lobectomy; NR: no tumor recurrence.

### Clinical Features

There were 6 females and 3 males in our study (median age 57.9 years, range 45–66 years). Of these 9 patients, 7 presented intermittent jaundice, which was the most common symptom. In this study, 6 patients had a history of relevant hepatobiliary surgery in other hospital. In the relevant surgery record, all patients were recorded with clinically detectable mucin after incision of common bile duct. Among these six patients, 4 cases received bile duct exploration and T-tube drainage without hepatectomy; and other two cases received cholecystectomy, left lateral hepatectomy, bile duct exploration and T-tube drainage. Two patients were admitted with T-tube, which still have mucin efflux on admission. Carbohydrate antigen 19-9 (CA 19-9) was within normal limits except for slight increase in two cases (22.2%). HBV infection was noted in two cases (22.2%). Other routine laboratory examination results were all within normal limits.

### Imaging Features

Abdominal ultrasonography showed various degrees of bile duct dilatation in all patients (measurements 21.3 ±14.2 mm). Six available CT scans showed varying degrees of bile duct dilatation, and found space-occupying liver lesions in five cases with poor enhancement. All patients were received preoperative MRCP. Magnetic resonance imaging demonstrated that stratification between mucus and bile was seen in five cases on T2 phase; asymmetry of bile duct dilatation in 6 cases; space-occupying lesions contrasted to high signal of bile in 4 cases. Above this finding, preoperative diagnostic specificity was 77.8% with two cases misdiagnosed of intrahepatic lithiasis.

### Surgical Findings and Treatment Outcome

In all patients, jelly-like substance could be seen after incision of common bile duct. The jelly-like substance was yellow-white at color in 8 cases, and dark green in one case. After removal of intraductal mucin, hilar bile duct was carefully explored. The jelly-like substance was eluted from the debouchement of diseased bile ducts, with more obvious after pressurizing the diseased side of liver. However, normal bile flow could be found in contralateral healthy side.

Among nine patients, 4 cases had papillary neoplasms at hilar bile duct, including 1 case at the bifurcation of left hepatic ducts, 2 cases at left hepatic ducts and involving the confluence, and 1 case at the bifurcation of left hepatic ducts in addition to the posterior wall of right hepatic ducts. The neoplasms were located at the left intrahepatic bile duct in other five patients. Left hemihepatectomy was carried out in all 9 patients to ensure 5-mm surgical margin adjacent to the tumor, including 2 cases who underwent the remnant left hemihepatectomy. Four cases were given anaplasty of hilar bile duct plus Rouxen-Y biliary-enteric anastomosis.

The average operative time was 194 min, and average blood loss was 633 ml. No postsurgical complication was noted. The mean postoperative hospital stay was 14 days (range from 8 to 28 days).

### Pathologic Finding and Follow-up

Mucin-producing bile duct tumors have a histological spectrum from low- and high-grade papillary growing to in situ and/or invasive carcinoma and sometimes to mucinous carcinoma [9]. Following tumor resection, permanent sections with hematoxylin and eosin staining were prepared.

Pathological examination of the surgical specimen revealed that neoplasms were located at intrahepatic bile duct in five cases, including serous cystadenoma in three cases, mucinous cystadenoma in two cases. In the other 4 patients, there was papillary adenocarcinoma in one case, papillary adenoma with local cancerization in one case, mucinous adenocarcinoma in one case, and tubular adenoma in one case. There was no tumor infiltration of surrounding tissue, no neural invasion, and no lymph node metastasis in all 9 patients.

One case was received ERCP (endoscopic retrograde cholangiopancreatography) to removing calculus in the common bile duct seven years after the surgery. One case was found intra-abdominal recurrence followed up to 59 months, who had been received two surgery with resection of ovarian mucinous cystadenoma and left hemihepatectomy. In this third surgery, two metastasis nodules, 9.0×4.0 cm and 7.0×3.0 cm in size, were found in the posterior wall of abdominal cavity. The mass was completely removed. At the time of writing, the patient is alive without disease recurrence 7 years after initial surgery. The remaining eight patients were alive with no recurrent during follow-up.

## Discussion

MPBTs are rare bile duct tumors that secrete clinically detectable mucin into bile duct [1]. The common presentation is obstructive jaundice and cholangitis secondary to biliary obstruction by mucin [2], [3], [10]. The tumor exhibits prominent papillary proliferation of neoplastic epithelia and shows various features of intraductal tumor growth. These tumors are often encountered in patients with inflammatory biliary lesions such as hepatolithiasis [7] and clonorchiasis [11].

Similar to IPMNs of pancreas, MPBTs have been considered to have relatively favorable prognosis after complete resection [10]. However, an appropriate surgical strategy has not ever been documented, partly because MPBTs are uncommon. There have been a few reports in literature providing details on patients with MPBTs [1], [10]. We analyzed the clinical records of 9 patients with MPBTs who underwent ipsilateral hemihepatectomy, and reported our findings here.

### Intermittent jaundice (jaundice history) is a major clinical feature of MPBTs

The tumor that has arisen from the mucosa may stay and attach to only a limited area of the bile ducts. It often spreads superficially along the mucosa and inner surface of the bile ducts. Even when the tumor spreads diffusely along a considerable length of the mucosa, the tumor does not show an invasive nature [12]. Many mucin-producing bile duct tumors are dormant in the bile duct for a considerable, asymptomatic period of time. Only in the late stage, an invasive carcinoma develops and shows a clinical course similar to that of cholangiocarcinoma in some cases [12], [13]. Therefore complete resection with good prognosis can be obtained as early diagnosis is identified.

The mucin varies is retained in the tumor cells or in the cysts in most cases. Mucobilia, a rare condition, is characterized by copious mucin secretion within the extrahepatic bile ducts, resulting in obstructive jaundice and cholangitis [14]. Although mucin secretion is a characteristic feature of MPBTs, profuse mucorrhea within the extrahepatic bile ducts is not often encountered [1], [10]. However, in our study all the patients were found to have mucobilia. This appears to impede the passage of bilirubin, resulting obstructive jaundice and cholangitis.

The tumors produce abundant mucin, sometimes in profuse amounts, and the mucin is excreted into bile ducts. The viscid mucin, in addition to the soft fragile tumor itself, fills the bile duct lumen, which causes partial obstruction and results in tubular dilatation of affected segmental or lobar bile ducts [15]. The bile ducts are rarely completely obstructed because the patency of the lumen is maintained between bile duct wall and papillary surfaces of intraductal tumors. Therefore, intermittent jaundice is different from progressive jaundice caused by gallbladder cancer invasion of extrahepatic bile duct or/and hilar cholangiocarcinoma.

### Preoperative assessment is the basis for achieving radical resection

Preoperatively, MPBTs can be definitely diagnosed by its characteristic histopathologic findings on biopsy specimens and proof of mucin production. However, there may be false negative or incorrect determination of tumor extent when the tumor is small. Therefore, multimodality assessment is contributed to correct diagnosis of MPBTs.

Various imaging studies, such as abdominal ultrasonography and computed tomography scans, were found to be helpful in the diagnosis of space-occupying liver lesions. The most characteristic appearance of MPBTs on computed tomography or ultrasonography scans is marked dilatation of the intra- and extrahepatic bile ducts distal to hepatic mass [2]. Unfurturately, in most cases, computed tomography and ultrasonography scans cannot differentiate mucin from bilirubin. However, echogenic spots in the dilated bile ducts on ultrasonography or highattenuation computed tomography are helpful in suggesting retention of mucin [2], [16].

Cholangiography is valuable for the detection of a mass and its extent when the bile ducts are well opacified. However, this modality has limited value when the bile ducts are not well opacified, especially when the opacification is hampered by the presence of large amount of mucin. When there is a small filling defect in the bile duct, it is difficult or impossible to differentiate small tumor from thick mucus ball.

Because excessive mucin in the bile ducts interferes with dye instillation and visualization, PTC and ERCP rarely contribute valuable diagnostic information.

In previous report, cholangiography following mucin drainage through PTBD is indispensable for obtaining accurate information about biliary anatomy and cancer extension [17]. Unfortunately, dislodgement of PTBD catheter and hemobilia are the common complications in these procedures. And the catheter should be flushed with saline daily to prevent obstruction of the catheter with viscous mucin. This greatly increase the difficulty of nurse care. In addition, there are invasive procedures, which may cause harm to patients. The procedures could also increase the risk of tumor metastasis in cases of malignant tumors [1], [18]. Therefore, we strongly recommend non-invasive imaging.

We emphasized the importance of MRCP for correct diagnosis of MPBTs in our analysis. In MRCP, the imaging features of MPBTs were significantly different from gallbladder carcinoma involing hilar bile duct and/or hilar cholangiocarcinoma.

**Figure 2:**
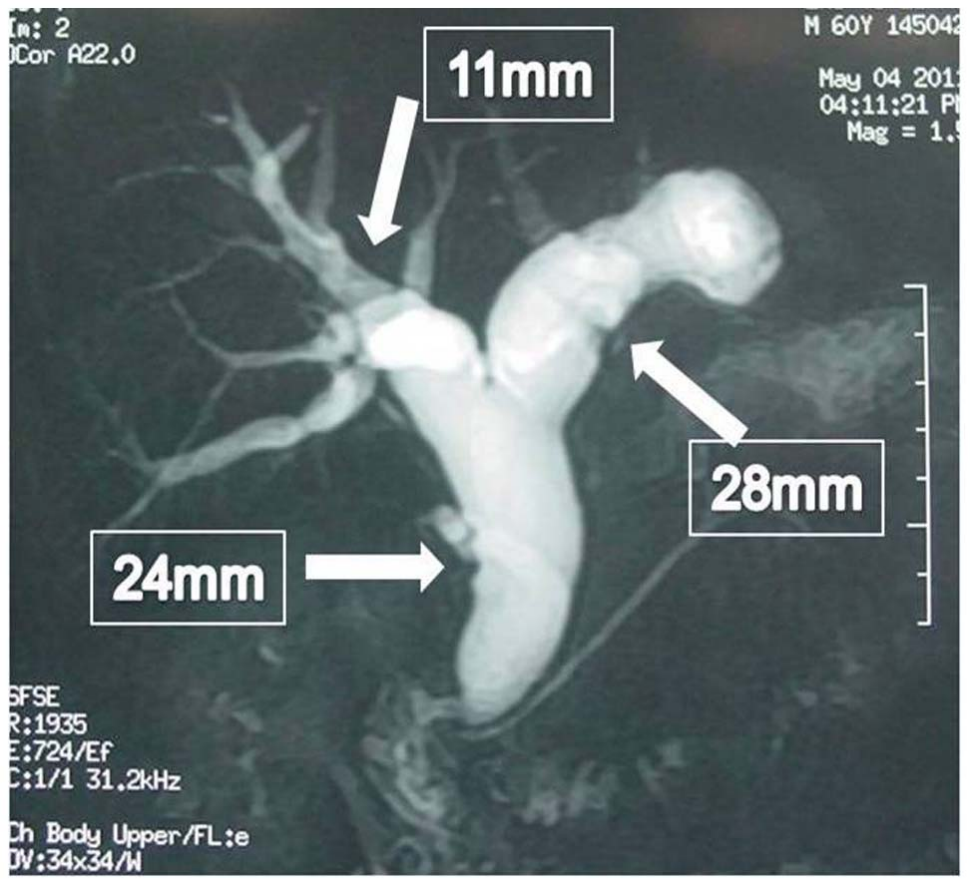
Asymmetry of bile duct dilatation. Shown: expansion in both extrahepatic and left intrahepatic bile ducts, with slight expansion of contralateral bile duct.

We summarized the imaging features on MRCP as follows: (1) on T2 phase, showing signs of stratification between mucus and bilirubin, so called “cascading sign”; (2) without significantly increased gallbladder, different from “turgescent gallbladder” in ampullary tumors; (3) no suddenly cut-off performance of the bile ducts as in other bile duct tumors; (4) asymmetry of bile duct dilatation, such as the significant expansion in both extrahepatic and ipsilateral intrahepatic bile ducts, with only slight expansion of contralateral bile duct (Fig. 2); (5) mass performance within bile duct contrasted to high signal of bilirubin in typical cases.

In our experience, whenever meeting the above three or more imaging features on MRCP and having intermittent jaundice, MPBTs must be highly suspected.

### Preoperative differential diagnoses

The differential diagnoses of MPBTs include biliary mucinous cystic neoplasm (MCN), mass-forming-type intrahepatic cholangiocarcinoma (ICC) with choledochal cysts, and recurrent pyogenic cholangitis with bile duct stones [19]. MPBTs lack ovarian-like stroma and communicate with the bile ducts, unlike biliary MCN. Meanwhile, unlike biliary MCN that is usually confined in a closed cyst, MPBTs can spread along the mucosal surface of the bile ducts.

On the other hand, malignant MPBTs is categorized as an intraductal-growth type of ICC. In comparison to other types of ICC, such as the mass-forming type and periductalinfiltrating type that have poor resectability and an unfavorable prognosis, malignant MPBTs can be completely resected and demonstrate more favorable prognosis [16].

The recurrent pyogenic cholangitis with bile duct stones causes intermittent and incomplete biliary obstruction and intraluminal masses or filling defects on images, similarly to those observed for MPBTs [16], [19]. Mucin plugs or sloughed masses of MPBTs may also be confused with bile duct stones. Therefore, recurrent pyogenic cholangitis with bile duct stones may be difficult to differentiate from MPBTs based on the analysis of imaging modalities alone. Regarding differentiation, invasive methods such as endoscopic cholangiography or cholangioscopy may be necessary to prove the presence of mucin plugs. In our study, two patients were both misdiagnosed as biliary lithiasis.

### The importance and outcome of ipsilateral hemihepatectomy in MPBTs

Unlike patients with IPMNs of pancreas, all patients with MPBTs should be treated, even if these tumors are regarded as benign, because mucin produced by MPBTs causes recurrent cholangitis and obstructive jaundice [1]. Because no treatment option other than surgical resection has been established to date, all MPBTs are recommended to be surgically resected as long as the patient is a good surgical candidate with a reasonable life expectancy.

In order to choose the appropriate surgical procedure, exact preoperative assessment of tumor location and cancer extension is important. In particular, to evaluate the extent of the spreading, observation of the mucus origin and tumor localization after complete mucin removal seems to be essential. However, the assessment of the invasion depth is not always easy unless MPBTs have invaded hepatic parenchyma or large vessels such as portal vein and hepatic artery, although intraductal ultrasonography may be useful for this assessment. Intraoperative frozen biopsy at the stumps of the bile duct is essential to confirm a cancer-free surgical margin. When precise diagnosis is completed preoperatively, the key of a successful operation is to determine the boundaries and scope of the tumor, and then completely resect it.

On the basis of preoperative assessments, the extent of hepatic resection and the point where the bile duct is to be divided should be planned. In principle, MPBTs should be resected in a manner similar to that employed for other types of bile duct carcers. That is, ipsilateral hemihepatectomy with or without extrahepatic bile duct resection (BDR) should be chosen as the surgical procedure. Even it is suspected that the tumor is an adenoma, a similar strategy should be considered, because MPBTs are often composed of varying degrees of cytoarchitectural atypia, and an accurate diagnosis of the maximum degree of cytoarchitectural atypia cannot be always made by preoperative biopsy. In fact, one MPBTs in our series, who was finally diagnosed as cancer, was preoperatively assessed as borderline adenoma. In our series, all the patients underwent ipsilateral hemihepatectomy with satisfactory prognosis.

### The failure reason for relevant hepatobiliary surgery in MPBTs

It is widely known that MPBTs are occasionally associated with hepatolithiasis [7], [20]. Bile stasis and repeated cholangitis may lead to the development of periductal inflammation, followed by biliary dysplasia, papillary hyperplasia with dysplasia, and in situ and invasive cholangiocarcinoma [7]. Mucobilia observed in patients with MPBTs may lead to the same inflammatory condition and adenoma-carcinoma sequence.

Unfortunately, many MPBTs patients had been followed for a long time before the correct diagnosis was obtained. Some were incorrectly diagnosed as congenital choledochal cysts or choledocholithiasis and treated by choledochostomy with T tube drainage or bilioenteric anastomoses before definitive diagnosis [1], [17]. Some patients underwent multiple surgeries for recurrent obstructive jaundice and cholangitis. Such delay of definitive diagnosis and biliary surgery without curative resection of primary lesion would make the patients' prognosis poor and add to the patients' financial burden.

Six patients with a history of relevant surgery in our group, were due to incomplete removal of the lesion, resulting in unalleviated postoperative symptoms. The main reason of reoperation was lack of adequate understanding of such diseases in initial surgery. When intraductal mucin was found during the operation, inexperienced surgeons failed to identify the extent of the lesion. Hence, insufficient hepatectomy is performed leaving remnant lesions.

When precise diagnosis was completed preoperatively and boundaries of the tumor infiltration was determined intraopratively, radical resection with long-term survival would be achieved. However, recent molecular biological studies suggested the correlations of the expression of MUC-1 and MUC-2 mucin antigens with the prognosis of intrahepatic bile duct tumors [21]. Patients with biliary tract malignancies who were MUC1-positive and MUC2-negative immunohistochemically showed poorer survival than MUC1-negative and MUC2-positive patients [22]. Further analyses need to be done to confirm the correlations of the expression of MUC-2 mucinantigen with a favorable prognosis in MPBTs.

In conclusion, intermittent jaundice is the most common manifestation of MPBTs, with typical characteristics on MRCP. Based on our follow up of 9 cases that were surgically treated for MPBTs, we conclude that ipsilateral hemihepatectomy is a safe surgical procedure with an observed recurrence risk of 11.1% and all long-term survival.
